# Microglia regulate brain progranulin levels through the endocytosis/lysosomal pathway

**DOI:** 10.1172/jci.insight.136147

**Published:** 2021-11-22

**Authors:** Tingting Dong, Leon Tejwani, Youngseob Jung, Hiroshi Kokubu, Kimberly Luttik, Terri M. Driessen, Janghoo Lim

**Affiliations:** 1 Department of Genetics, Yale School of Medicine, New Haven, Connecticut, USA.; 2 Interdepartmental Neuroscience Program, Yale University, New Haven, Connecticut, USA.; 3 Department of Neuroscience,; 4 Program in Cellular Neuroscience, Neurodegeneration and Repair, and; 5 Yale Stem Cell Center, Yale School of Medicine, New Haven, Connecticut, USA.

**Keywords:** Neuroscience, Dementia, Mouse models, Neurodegeneration

## Abstract

Genetic variants in *Granulin* (*GRN*), which encodes the secreted glycoprotein progranulin (PGRN), are associated with several neurodegenerative diseases, including frontotemporal lobar degeneration, neuronal ceroid lipofuscinosis, and Alzheimer’s disease. These genetic alterations manifest in pathological changes due to a reduction of PGRN expression; therefore, identifying factors that can modulate PGRN levels in vivo would enhance our understanding of PGRN in neurodegeneration and could reveal novel potential therapeutic targets. Here, we report that modulation of the endocytosis/lysosomal pathway via reduction of Nemo-like kinase (Nlk) in microglia, but not in neurons, can alter total brain Pgrn levels in mice. We demonstrate that Nlk reduction promotes Pgrn degradation by enhancing its trafficking through the endocytosis/lysosomal pathway, specifically in microglia. Furthermore, genetic interaction studies in mice showed that *Nlk* heterozygosity in *Grn* haploinsufficient mice further reduces Pgrn levels and induces neuropathological phenotypes associated with PGRN deficiency. Our results reveal a mechanism for Pgrn level regulation in the brain through the active catabolism by microglia and provide insights into the pathophysiology of PGRN-associated diseases.

## Introduction

Progranulin (PGRN) is an evolutionarily conserved, cysteine-rich, secreted glycoprotein encoded by the *Granulin* (*GRN*) gene (1, 2). In humans, several neurodegenerative diseases are closely linked to the reduction of PGRN expression. Haploinsufficiency of PGRN, caused by heterozygous loss-of-function mutations in 1 *GRN* allele, leads to the development of autosomal dominant familial frontotemporal lobar degeneration (FTLD), a neurodegenerative disease characterized by atrophy of the frontal and temporal lobes of the brain (2–6). Homozygous loss-of-function mutation of both *GRN* alleles causes neuronal ceroid lipofuscinosis (NCL), a lysosomal storage disease in which lipofuscins aberrantly accumulate in the affected tissues (7). Finally, specific single nucleotide variants in *GRN* have been shown to decrease plasma and brain expression levels of PGRN and are risk factors for Alzheimer’s disease (AD), the most common form of dementia (8–10).

Although the involvement of *GRN* genetic variants in human disease appears to be straightforward, the precise effect of changes to *Grn* in animal models is far more elusive. In mice, *Grn* heterozygosity induces limited changes in behavior and neuropathology up to 23 months of age (11), which include age-dependent social and emotional deficits (12), as well as lysosomal abnormalities (13). In contrast, mice lacking both copies of *Grn* display many behavioral and neuropathological abnormalities, which recapitulate several hallmark features of PGRN-deficient FTLD (FTLD-PGRN) and NCL (12, 14–16). Furthermore, Pgrn has been shown to be a modulator in animal models of neurodegenerative diseases including AD (9, 17) and Parkinson’s disease (18). Together, these studies demonstrate that decreased expression of PGRN is a direct cause, or indirect risk factor, for a variety of neurodegenerative diseases. Interestingly, conditional deletion of *Grn* in neurons and/or microglia is not sufficient to induce the neuropathological changes associated with FTLD, suggesting that reduced production of Pgrn in one cell type can be compensated by non-cell-autonomous Pgrn production in other cells (19–21).

The mechanisms through which reductions in central nervous system PGRN levels lead to diverse neurodegeneration phenotypes remain a largely unanswered question in the field. Previous works suggest that Pgrn plays important roles in the regulation of neurite growth, innate immunity, and lysosome function, all of which are cellular processes studied extensively in the context of neurodegeneration (15, 22–28). More specifically, Pgrn can modulate the Wnt and Notch signaling pathways, which regulate axon regeneration, neuronal differentiation, and neuronal survival (29–31). Additionally, Pgrn plays a role in the regulation of neuroinflammation as an antiinflammatory factor (22, 24, 32), as loss of Pgrn leads to lysosomal defects and excessive complement production, triggering selective synaptic pruning by microglia (26). Several other studies have also highlighted the active roles of Pgrn in the control of lysosome formation and function (15, 27, 28) and that lysosomal Pgrn in neurons can protect against excitotoxicity (33).

Pgrn is expressed in a wide range of tissues and cell types, including neurons and activated microglia in the brain (34). Because Pgrn is a secreted molecule, several studies have focused on Pgrn trafficking and catabolism. Previous studies have demonstrated that extracellular Pgrn can undergo endocytosis through Sortilin receptor–dependent or –independent mechanisms, followed by subsequent delivery to the lysosome for processing in neurons (35, 36). How different cell types are involved in the process of Pgrn breakdown to regulate overall levels in the brain is a topic of interest within the field. Although previous studies have described several molecules that are critically involved in the regulation of Pgrn levels in neurons (15, 28, 35, 36), similar studies focusing on other brain cell types have not yet been performed. Thus, identifying factors capable of modulating the functioning and/or expression levels of Pgrn itself in other cell types is essential, as knowledge of such regulatory factors will not only impart invaluable information regarding the underlying pathophysiology of PGRN-associated neurodegenerative diseases but also uncover novel putative targets for therapeutic intervention.

Nemo-like kinase (Nlk) is an evolutionarily conserved serine/threonine kinase that plays roles in various signaling pathways, including Notch, Wnt, and DNA damage response pathways (37–42). Several lines of evidence suggest that Nlk could act as a potential protein to modulate the pathogenesis of PGRN-associated neurological diseases and the expression levels of PGRN in the brain. First, changes in Nlk and Pgrn levels induce similar cellular phenotypes. For example, both Nlk and Pgrn regulate neurite outgrowth (23, 25, 43) and neuroinflammation (2, 22, 24, 44). Second, Nlk and Pgrn influence similar molecular pathways that may underlie shared cellular functions. Both Nlk and Pgrn play a role in the modulation of Wnt and Notch signaling pathways, which are important for neuronal survival and axon regeneration (29–31, 37–41). Third, deletion of a component in the Nlk-mediated molecular signal transduction pathways leads to neuropathological phenotypes that are similar to AD and FTLD-PGRN (45). Specifically, Nlk-mediated phosphorylation of Nurr1 is essential for the prevention of neuroinflammation by inhibiting neurotoxic gene expression in microglia via the recruitment of CoREST/lysine-specific demethylase 1 (LSD1) complex for the transrepression of NF-κB activity (44), and mice lacking the histone demethylase *Lsd1* display behavioral, neuropathological, and molecular phenotypes that highly overlap with those seen in human AD and FTLD-PGRN cases (45).

In this study, we investigated the potential role of microglia in modulating Pgrn expression and function by using mouse models and cultured cells in which the activity of the endocytosis/lysosomal pathway was altered by changing Nlk levels. Genetic interaction studies in mice demonstrated that Nlk functions as a positive regulator of brain Pgrn levels and that loss of Nlk leads to a reduction in Pgrn levels. Furthermore, in vitro and in vivo approaches demonstrated Nlk-mediated regulation of Pgrn levels in the brain occurs through microglia by controlling clathrin-dependent Pgrn endocytosis and delivery to the lysosome. Finally, Grn heterozygote mice display FTLD-like neuropathological abnormalities in an Nlk heterozygote background at 1 year of age. These results reveal a mechanism for the regulation of Pgrn levels in the brain and provide insight into the pathophysiology of several neurodegenerative diseases associated with PGRN expression levels, such as AD, FTLD, and NCL.

## Results

### Decreased expression of Nlk reduces Pgrn levels in vivo.

**Figure 1:**
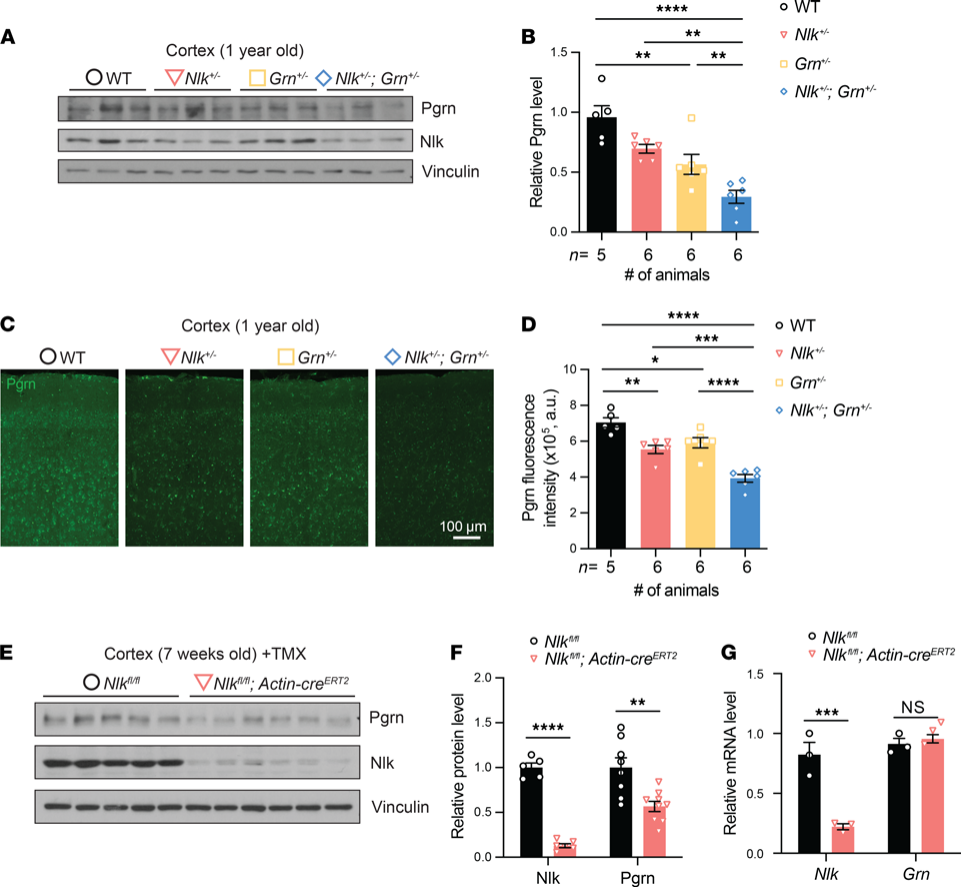
Loss of Nlk regulates Pgrn levels in the mouse cortex. (**A–D**) Expression levels of Pgrn were significantly decreased in the mouse cortex of *Nlk^+/–^ Grn^+/-^* mice compared with their littermate controls. Representative Western blot images (**A**) and quantification (**B**) of Pgrn and Nlk expression in the 1-year-old mouse cortex. Normalized protein levels of Nlk and Pgrn signals to Vinculin are shown in this and all following graphs. Error bars represent standard error of the mean (SEM) in this and all following graphs. **P < 0.01, ****P < 0.0001; 1-way ANOVA with Tukey’s multiple comparisons post hoc test; *F*(3,19)=15.45, *P* < 0.0001. Representative confocal images (**C**) and quantification (**D**) of Pgrn immunofluorescent staining in a 1-year-old mouse cortex. Total fluorescence intensity quantification across the image field was automated using Volocity software. *P < 0.05, **P < 0.01, ***P < 0.001, ****P < 0.0001; 1-way ANOVA with Tukey’s multiple comparisons post hoc test; *F*(3,19)=26.11, *P* < 0.0001. (**E–G**) Temporal deletion of Nlk in all cell types reduces Pgrn protein levels in the mouse cortex. Protein and mRNA expression levels were analyzed in the 7-week-old cortex of *Nlk^fl/fl^* and *Nlk^fl/fl^ Actin-cre^ERT2^* mice after tamoxifen (TMX) injection. Representative Western blot images (**E**) and quantification (**F**) showing the reduced Nlk and Pgrn expression in the mouse cortex. **P < 0.01, ****P < 0.0001 (nonparametric Mann-Whitney *t* test, *n* = 8 animals for *Nlk^fl/fl^*, *n* = 9 for *Nlk^fl/fl^ Actin-cre^ERT2^*). (**G**) Quantification of *Grn* mRNA expression levels in *Nlk*-deleted mouse cortex, showing no transcriptional effects. Normalized levels of *Nlk* and *Grn* mRNA to mouse *ACTB* are shown in this and all following graphs. ***P < 0.001, NS, nonsignificant (nonparametric Mann-Whitney *t* test, *n* = 3). In this and all following figures, mouse genotypes are color-coded. Black represents WT, pink for *Nlk^+/–^*, yellow for *Grn^+/–^*, and blue for *Nlk^+/–^ Grn^+/–^* unless otherwise mentioned.

We have recently identified Nlk as a negative regulator of the lysosome in neurons (46). Therefore, because Pgrn is trafficked to and degraded by the lysosome (35, 36, 47), we wondered whether partial loss of *Nlk* alters the levels of Pgrn. We found that the Pgrn protein was significantly reduced in the cortex of 1-year-old *Nlk^+/–^ Grn^+/–^* mice compared with *Grn^+/–^* littermates (Figure 1, A and B). Immunofluorescence staining for Pgrn in the mouse cortex also showed a decrease in Pgrn intensity in *Nlk^+/–^ Grn^+/–^* relative to *Grn^+/–^*, and a decrease in *Nlk^+/–^* relative to wild-type (WT) controls (Figure 1, C and D). These data suggest that Nlk positively regulates Pgrn expression in vivo.

To determine if a complete loss of *Nlk* enhances the downregulation of Pgrn, we temporally deleted *Nlk* in all cell types during adulthood to circumvent *Nlk*-knockout (*Nlk*-KO) prenatal lethality (48). We took advantage of mice with a flox-*Nlk* allele (49) and crossed them to mice expressing tamoxifen-inducible (TMX-inducible) Cre driven by a ubiquitous CMV-IE enhancer and chicken β-actin promoter (50). We generated *Nlk^fl/fl^ Actin-cre^ERT2^* mice and their littermate mice on a pure C57BL/6J background. After intraperitoneal TMX injection for 7 consecutive days starting at 6 weeks of age, we verified that the expression levels of *Nlk* mRNA and protein were significantly reduced in the cortex of *Nlk^fl/fl^ Actin-cre^ERT2^* mice (Figure 1, E–G). Furthermore, there was a significant reduction of Pgrn protein levels in the cortex of *Nlk^fl/fl^ Actin-cre^ERT2^* mice compared to their littermates (Figure 1, E and F). Interestingly, serum levels of Pgrn were not altered (data not shown) upon TMX-induced conditional deletion of Nlk in all cell types, suggesting that Nlk-mediated regulation of Pgrn levels is restricted to the central nervous system and is not a globally occurring phenomenon.

The downregulation of Pgrn protein expression in *Nlk^fl/fl^ Actin-cre^ERT2^* mice may be due to a similar downregulation at the transcriptional level. To determine if *Grn* mRNA was also downregulated in *Nlk^fl/fl^ Actin-cre^ERT2^* mice, we utilized quantitative real-time reverse transcription polymerase chain reaction (qRT-PCR) to assess *Grn* expression in the cortex of 7-week-old mice. *Grn* mRNA level was not altered by *Nlk* deletion (Figure 1G). Together, these studies indicate that Nlk regulates Pgrn expression in the adult brain posttranscriptionally.

### Nlk regulates Pgrn levels via microglia, but not through neurons.

**Figure 2:**
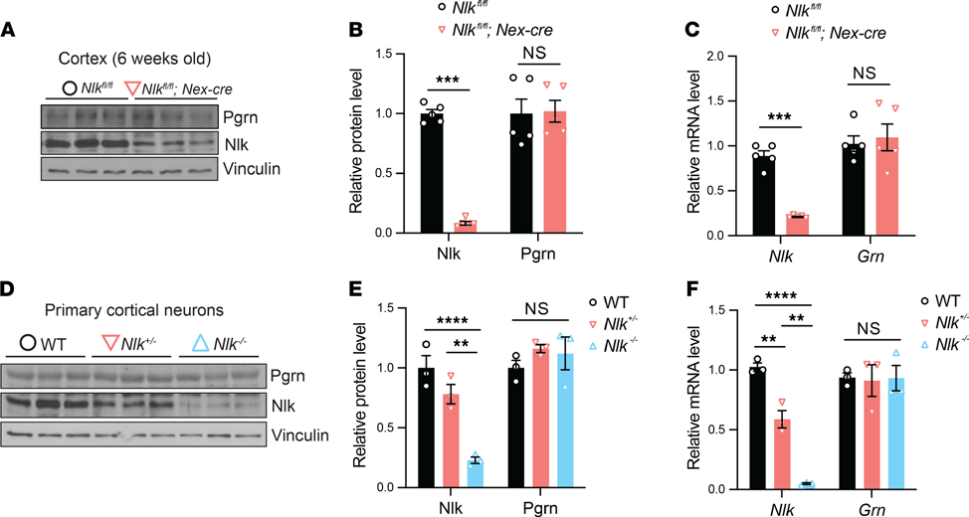
Nlk does not regulate Pgrn levels in neurons. (**A–C**) Nlk does not regulate Pgrn levels through neurons in vivo. Protein and mRNA expression levels were analyzed in the 6-week-old cortex of *Nlk^fl/fl^* and *Nlk^fl/fl^ Nex-cre* mice. Representative Western blot images (**A**) and quantification (**B** and **C**) showing that the expression levels of Pgrn protein or *Grn* mRNA in the whole cortex were not altered by Nlk deletion specifically in the principal neurons. ***P < 0.001 (nonparametric Mann-Whitney *t* test, *n* = 5). (**D–F**) Nlk does not regulate Pgrn levels in primary neurons in vitro. Expression levels of Nlk and Pgrn were analyzed in cultured primary cortical neurons from WT, *Nlk^+/–^*, and *Nlk^–/–^* mice. Representative Western blot images (**D**) and quantification (**E** and **F**) showing that the expression levels of Pgrn or *Grn* mRNA were not altered by Nlk expression levels in neurons. As previously reported (48), a small amount of residual Nlk remained (~10%) in *Nlk^–/–^* animals due to limitations of gene trap technology. **P < 0.01, ****P < 0.0001; 1-way ANOVA with Tukey’s post hoc testing, *n* = 3; *Nlk F*(2,6)=109.8, *P* < 0.0001; *Grn F*(2,6)=0.01789, *P* = 0.9823.

Since we observed that constitutive deletion of a single *Nlk* allele (Figure 1, A–D) or temporally induced deletion of both copies of *Nlk* in all cell types during adulthood (Figure 1, E–G) leads to a reduction of Pgrn protein expression in the adult mouse cortex, we next wanted to determine which cell types are involved in the Nlk-mediated regulation of Pgrn expression. Because *Nlk* and *Grn* are expressed in both neurons and glia in the cortex (34), we first tested whether Nlk-mediated regulation of Pgrn occurs in neurons by generating *Nlk^fl/fl^ Nex-cre* mice and their littermate controls on a pure C57BL/6J background. *Nex-cre* mice express Cre recombinase in the principal neurons of the cortex and the hippocampus, which persists through adulthood (51). The expression levels of *Nlk* mRNA and protein were significantly reduced in the cortex of *Nlk^fl/fl^ Nex-cre* mice at 6 weeks of age (Figure 2, A–C). Contrary to results from animals in which *Nlk* was partially or completely ablated in all cell types, no significant alterations in the expression levels of Pgrn protein or its mRNA were observed in the cortex of *Nlk^fl/fl^ Nex-cre* mice in vivo (Figure 2, A–C).

Since *Nex-cre* is expressed mainly in excitatory neurons, but not in inhibitory interneurons (51), we assayed Pgrn expression in constitutive *Nlk^+/–^* and *Nlk^–/–^* primary cortical neuron culture, which contains both excitatory and inhibitory neurons (52). There was no difference in Pgrn protein or mRNA expression levels between WT, *Nlk^+/–^*, and *Nlk^–/–^* cortical neurons in vitro (Figure 2, D–F). Taken together, these data suggest that the Nlk-mediated Pgrn regulation observed in vivo may not be occurring through neurons.

**Figure 3:**
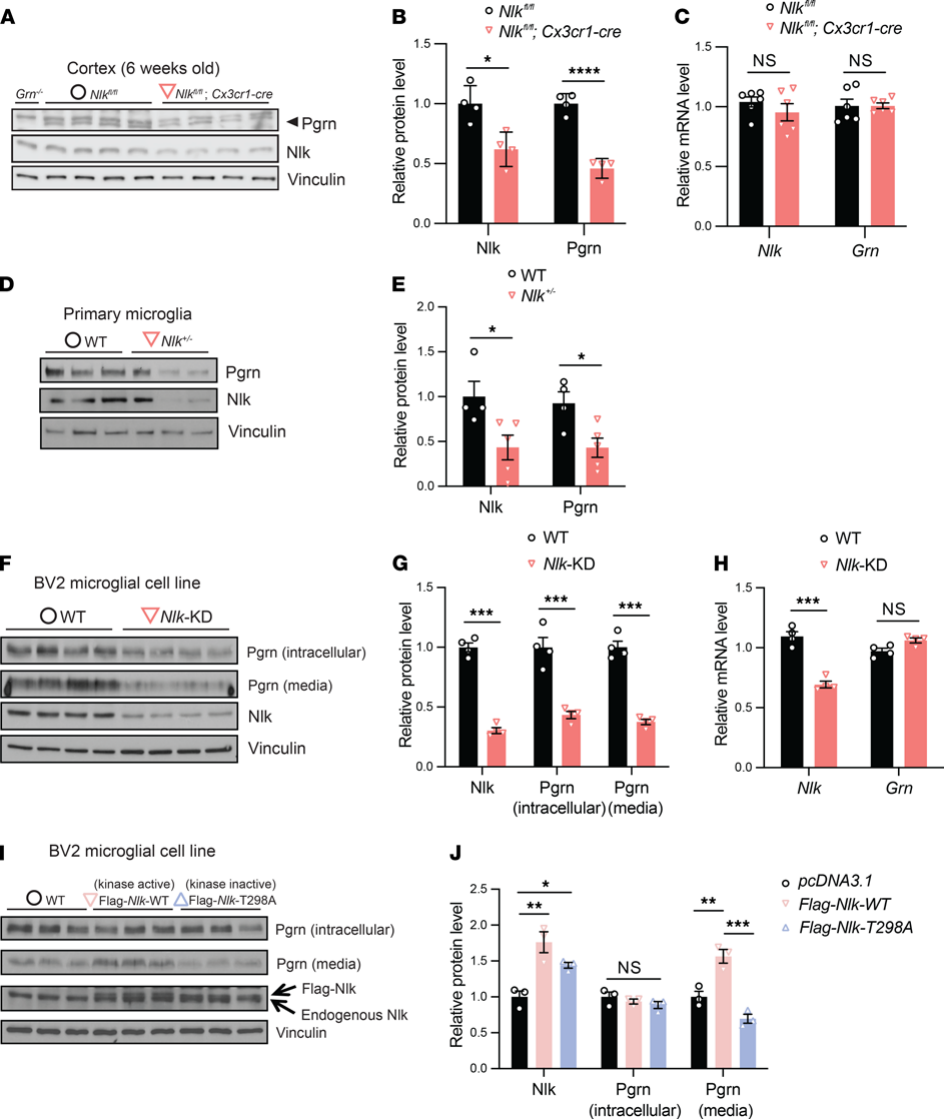
Nlk regulates Pgrn levels in microglia in a kinase activity-dependent manner. (**A–C**) Nlk regulates Pgrn levels in microglia in vivo. Protein and mRNA expression levels were analyzed in the 6-week-old cortex of *Nlk^fl/fl^* and *Nlk^fl/fl^ Cx3cr1-cre* mice. Representative Western blot images (**A**) and quantification (**B**) showing that the protein expression levels of Pgrn are significantly decreased by Nlk deletion in microglia. *Grn*^–/–^ is shown as a negative control. *P < 0.05, ****P < 0.0001 (nonparametric Mann-Whitney *t* test, *n* = 4–6 animals per group). Quantification (**C**) of *Nlk* and *Grn* mRNA expression levels in the cortex from microglia-specific *Nlk* deletion mice. NS, nonsignificant (nonparametric Mann-Whitney *t* test, *n* = 6). (**D** and **E**) Reduced Nlk expression significantly decreased Pgrn expression levels in primary microglia. Representative Western blot images (**D**) and quantification (**E**) of Nlk and Pgrn expression levels in primary microglia at DIV-16 from WT and *Nlk^+/–^* mice. *P < 0.05 (nonparametric Mann-Whitney *t* test, *n* = 5). (**F–H**) Nlk reduction resulted in decreased Pgrn levels in BV2 microglial cells. Protein and mRNA expression levels were analyzed in WT and *Nlk-*KD BV2 cells. Representative Western blot images (**F**) and quantification (**G** and **H**) showing the expression levels of Nlk and Pgrn proteins and their corresponding mRNAs. ***P < 0.001 (nonparametric Mann-Whitney *t* test, *n* = 4). (**I** and **J**) Increased expression of Nlk upregulated Pgrn levels in the media in a kinase activity–dependent manner in BV2 microglial cells. Representative Western blot images (**I**) and quantification (**J**) of Nlk and Pgrn levels in BV2 cells by Nlk overexpression. *Nlk*-T298A is a kinase-inactive form of *Nlk*. *P < 0.05, **P < 0.01, ***P < 0.001; 1-way ANOVA with Tukey’s post hoc testing; *n* = 3; Nlk *F*(2,6)=14.91, *P* = 0.0047; Pgrn (intracellular) *F*(2,6)=1.190, *P* = 0.3670; Pgrn (media) *F*(2,6)=30.16, *P* = 0.0007.

Transcriptomics studies have indicated that *Grn* is expressed highly in microglia (34). To test if microglia could be the primary cell type in Nlk-mediated Pgrn regulation, we generated *Nlk^fl/fl^ Cx3cr1-cre* mice and their littermate control mice on a pure C57BL/6J background. *Cx3cr1-cre* mice express Cre recombinase in microglia in the brain (53). We measured Pgrn expression by immunoblotting with a Pgrn antibody whose specificity we have confirmed using *Grn^–/–^* protein lysates (Figure 3A). We found that the expression levels of Pgrn protein were significantly reduced in *Nlk^fl/fl^ Cx3cr1-cre* mice compared to littermate controls at 6 weeks of age (Figure 3, A and B). Consistent with results from *Nlk^fl/fl^ Actin-cre^ERT2^* mice (Figure 1G), there was no significant change in *Grn* mRNA in *Nlk^fl/fl^ Cx3cr1-cre* mice compared to littermate controls (Figure 3C). To further confirm the role of microglia in Nlk-mediated regulation of Pgrn levels, we also performed primary microglia culture experiments from cortices of WT and *Nlk^+/–^* mice and monitored Pgrn expression. Consistent with our finding in vivo, *Nlk* haploinsufficiency resulted in a marked reduction of Pgrn levels in primary microglia culture in vitro (Figure 3, D and E).

Since loss of *Nlk* causes a downregulation of Pgrn, increasing *Nlk* expression should in turn elevate Pgrn expression. To test this, we utilized a murine microglial cell line, BV2. To first verify that BV2 cells recapitulate Nlk-mediated regulation of Pgrn as seen in primary microglia, we generated Nlk-deficient cells using CRISPR/Cas9 (54). Due to the additional chromosomes that result from the nondiploid nature of the BV2 cancer cell line, incomplete *Nlk* gene-targeted BV2 cell clones were identified, and 1 clone was selected as *Nlk*-knockdown (*Nlk*-KD) cells for our analyses, which led to a consistent knockdown efficiency of Nlk (about 70% reduction) at the protein level (Figure 3, F and G) and a significant reduction at the mRNA level (Figure 3H). Consistent with our results from primary microglia (Figure 3, D and E), there was a significant reduction in intracellular and media/extracellular Pgrn in *Nlk*-KD BV2 cells (Figure 3, F and G), and no change in *Grn* mRNA (Figure 3H), validating BV2 cells as a model for identifying the mechanism underlying the effect of Nlk on Pgrn levels.

To determine if overexpression of Nlk, and the kinase activity of Nlk, affects the regulation of Pgrn expression, we transiently transfected Flag-tagged *Nlk* and examined the expression levels of Pgrn protein in BV2 cells (Figure 3, I and J). Interestingly, increased expression of WT Nlk (Nlk-WT) strongly increased Pgrn levels in the extracellular media, but not in intracellular lysates, in BV2 cells (Figure 3, I and J). In contrast, kinase-inactive Nlk (Nlk-T298A) had no effect on the regulation of Pgrn in BV2 cells. T298A is a threonine to alanine substitution at residue 298 in the catalytic domain of Nlk, leading to defective kinase activity. Taken together, these data strongly suggest that Nlk deficiency decreases, and its overexpression increases, Pgrn levels in a kinase activity–dependent manner in microglia both in vivo and in vitro.

### Nlk controls Pgrn endocytosis in microglia in a clathrin-dependent manner.

**Figure 4:**
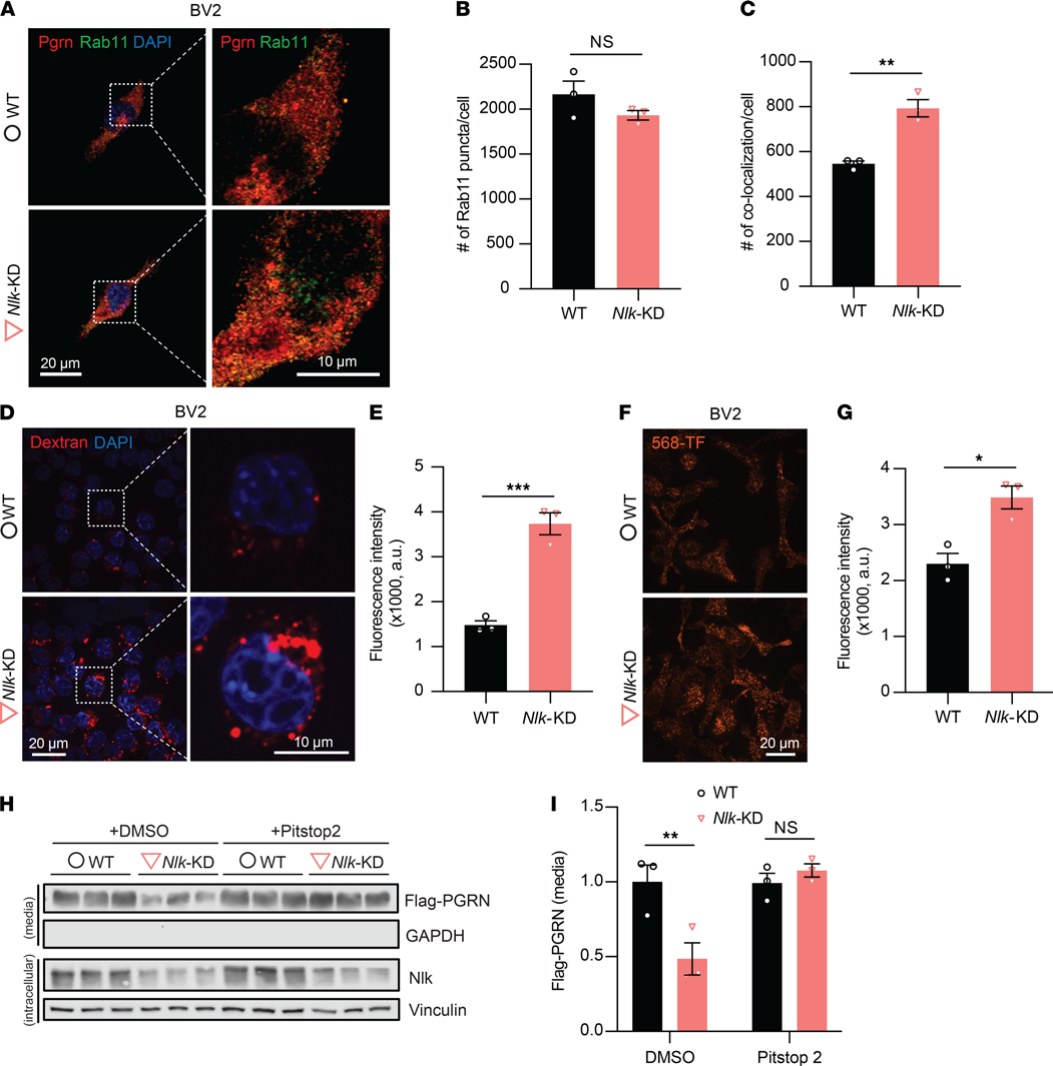
Nlk deficiency increases clathrin-dependent Pgrn endocytosis in microglia. (**A**) Nlk reduction resulted in the increased localization of Pgrn to endosomes. Representative images of WT and *Nlk-*KD BV2 cells costained for Pgrn/Rab11 demonstrated colocalization of Pgrn with recycling endosomes. Right panels are magnified views of areas marked in the left panels. (**B** and **C**) Quantification of Rab11 puncta (**B**) and colocalization Pgrn and Rab11 (**C**) are shown. **P < 0.01 (2-tailed, unpaired Student’s *t* test, *n* = 3 wells, average of ~10 cells sampled per well). (**D** and **E**) Enhanced uptake of the extracellularly provided dextran in *Nlk*-KD BV2 cells. Representative images (**D**) and quantification (**E**) of WT and *Nlk*-KD BV2 cells after 20 minutes’ incubation with 647-dextran. ***P < 0.001 (2-tailed, unpaired Student’s *t* test, *n* = 3 wells, average of ~50 cells sampled per well). (**F** and **G**) Enhanced uptake of the extracellularly provided transferrin in *Nlk*-KD BV2 cells. Representative images (**F**) and quantification (**G**) of WT and *Nlk*-KD BV2 cells after 15 minutes’ incubation with Alexa 568–transferrin. *P < 0.05 (2-tailed, unpaired Student’s *t* test, *n* = 3). (**H** and **I**) Representative Western blot images (**H**) and quantification (**I**) showing the enhanced clathrin-dependent endocytosis of Flag-tagged PGRN provided exogenously in BV2 microglial cells. Cells were treated with DMSO or the clathrin-dependent endocytosis inhibitor Pitstop 2 for 1 hour and incubated with the recombinant Flag-PGRN (1 nM) for 15 minutes. **P < 0.01; 2-way ANOVA with post hoc Bonferroni correction, *n* = 3; *F*(1,8)=11.85, *P* = 0.0088.

We next sought to elucidate the cellular mechanism through which Nlk regulates Pgrn expression levels. We first examined the specific subcellular localization of Pgrn in WT and *Nlk-*deficient cells, using both BV2 and primary microglia. We found that intracellular punctate Pgrn signal was higher in *Nlk*-KD BV2 cells and *Nlk^+/–^* primary microglia compared to WT controls (Supplemental Figure 1, A–D; supplemental material available online with this article; https://doi.org/10.1172/jci.insight.136147DS1), suggesting vesicular trafficking of Pgrn along the endocytosis/lysosomal pathway may be higher, since Pgrn is known to colocalize with lysosome-associated membrane protein 1 (Lamp1) (27, 35). To test this idea, we costained for Pgrn and several lysosome and endosome markers in WT and *Nlk*-KD BV2 cells. As expected, Pgrn partially colocalized with Lamp1 (lysosome), Rab5 (early endosome), Rab7 (late endosome), and Rab11 (recycling endosome) (Figure 4, A–C, and Supplemental Figure 2). Colocalization of Pgrn with recycling (Rab11) endosomes was increased in *Nlk*-KD cells compared with WT cells (Figure 4, A–C), suggesting that Nlk may mediate trafficking of Pgrn along the endosome/lysosome pathway. Due to the lack of available antibodies for immunofluorescence detection of Nlk, we were not able to demonstrate the subcellular cocompartmentalization of endogenous Nlk protein to similar intracellular structures as Pgrn.

Collectively, the knowledge of the high degree of colocalization of Pgrn with endosomal markers in Nlk-KD cells (Figure 4, A–C), the enhanced Pgrn levels in extracellular media following Nlk overexpression in BV2 cells (Figure 3, I and J), and the fact that Pgrn is a secreted protein that can be endocytosed and delivered to lysosomes (35) raise the possibility that Nlk regulates microglial endocytosis. Thus, we decided to test whether Nlk functionally affects endocytic processes in microglia. To do so, WT and *Nlk*-KD BV2 microglial cells were incubated with fluorescently labeled dextran. As expected, Nlk depletion significantly enhanced fluorescently labeled dextran uptake by BV2 cells (Figure 4, D and E). We also found a similar effect using the fluorescently labeled transferrin in *Nlk*-KD BV2 microglial cells (Figure 4, F and G) and primary microglia from *Nlk^+/–^* mice (Supplemental Figure 3, A and B). To determine if Nlk regulates endocytosis in neurons as well, primary cortical neurons were cultured from WT, *Nlk^+/–^*, and *Nlk^–/–^* mice and transferrin uptake was examined. In stark contrast to our results from BV2 microglial cells and primary microglia, *Nlk* deletion did not affect the uptake of fluorescently labeled transferrin in primary neurons (Supplemental Figure 3, C and D), indicating that Nlk reduction enhances endocytosis of multiple cargoes in microglia, but not in neurons.

Next, to test directly if Nlk affects microglial endocytosis specifically, we treated WT and *Nlk*-KD BV2 microglial cells with exogenous Flag-tagged recombinant PGRN proteins. We measured Flag-tagged PGRN proteins remaining in the media after a 15-minute incubation and found a significantly decreased level of Flag-tagged PGRN remaining in the medium from the *Nlk*-KD BV2 cells compared with that from WT controls (Figure 4, H and I). Since endocytosis can be divided into phagocytosis, pinocytosis, and receptor-mediated endocytosis, we investigated which specific pathway is likely regulated by Nlk. Using Pitstop 2, a specific inhibitor of clathrin-mediated endocytosis, we found that the Nlk-regulated endocytosis of Flag-tagged PGRN was completely blocked (Figure 4, H and I). Although we cannot exclude the possibility that Nlk may also regulate the exocytosis of PGRN, these data, along with the data showing increased uptake of fluorescently tagged dextran and transferrin, suggest that Nlk plays a role in receptor-mediated endocytosis of PGRN.

### Microglial Nlk-mediated regulation of Pgrn levels is dependent on lysosomal degradation.

**Figure 5:**
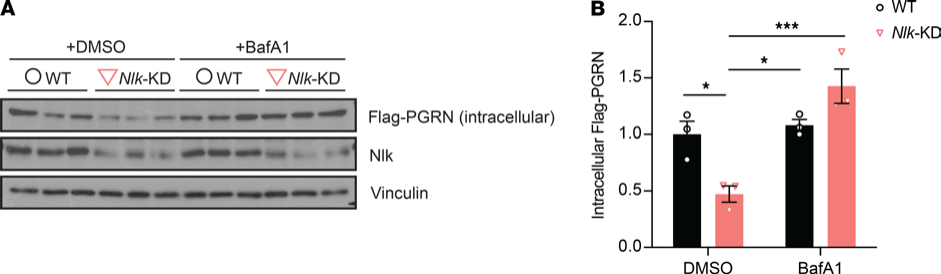
Microglial Nlk-mediated regulation of Pgrn levels is dependent on lysosomal degradation. (**A** and **B**) Representative Western blot images (**A**) and quantification (**B**) showing the enhanced degradation of the exogenously provided recombinant Flag-PGRN protein in *Nlk*-KD BV2 cells, which is lysosome activity dependent. Cells were treated with DMSO or BafA1 for 4 hours and incubated with the recombinant Flag-PGRN (20 nM) for 15 minutes. *P < 0.05, ***P < 0.001; 2-way ANOVA, *n* = 3; *F*(1,8)=17.12, *P* = 0.0033.

Considering the evidence that Nlk regulates Pgrn uptake via endocytosis in BV2 cells (Figure 4, H and I), it is likely that Pgrn is then processed via lysosome-dependent degradation. To reconcile the fact that Nlk reduction promotes microglial Pgrn endocytosis and that we observe a reduction of Pgrn protein levels in *Nlk^+/–^ Grn^+/–^* compound heterozygote animals (Figure 1, A–D), mice with conditional microglial deletion of Nlk (Figure 3, A and B), Nlk-deficient primary microglia (Figure 3, D and E), and BV2 cells (Figure 3, F and G), we assessed the involvement of Nlk-mediated lysosomal degradation of Pgrn. We treated *Nlk*-KD and WT BV2 cells with the lysosome inhibitor Bafilomycin A1 (BafA1), which blocks lysosome-mediated degradation by inhibiting vATPase-mediated acidification of ysosomes, and examined levels of Pgrn. Biochemical analyses showed that Nlk deficiency–mediated degradation of exogenously provided Flag-tagged PGRN (Figure 5, A and B) was indeed dependent on lysosomal activity in BV2 microglial cells. Taken together, these studies strongly suggest that Nlk regulates Pgrn levels in the brain in a lysosome-dependent manner through microglia.

### Decreased Nlk expression induces neuropathological phenotypes in heterozygous Grn^+/–^ mice.

Previous studies have documented that *Grn^+/–^* heterozygous mice display mild social and lysosomal abnormalities (13, 55); however, these animals do not display any overt gliosis, neurodegeneration, or lipofuscin accumulation, all neuropathological phenotypes associated with *GRN* haploinsufficiency in humans (11, 56, 57). In contrast, *Grn^–/–^* null mice exhibit mild to moderate cognitive impairment and pathological abnormalities at 12–18 months of age (16, 58, 59). Although *Grn^+/–^* mice are phenotypically only mildly affected, it is possible that *Grn^+/–^* mice may exhibit a robust synergistic phenotype in certain genetic backgrounds, such as with reduced Nlk expression, which further reduces Pgrn levels in vivo (Figure 1). To explore the possibility that the neuropathological phenotypes associated with loss of *Grn* are affected by Nlk, we conducted a genetic interaction study using mouse models of *Grn* and *Nlk*. We crossed *Grn^+/–^* mice with *Nlk^+/–^* animals and evaluated the progeny on a pure C57BL/6J background, investigating whether *Nlk^+/–^ Grn^+/–^* double heterozygote mice could develop pathological and behavioral changes.

We first investigated whether a 50% reduction of Nlk expression induces pathological phenotypes in *Grn^+/–^* mice. Previous studies have demonstrated that *Grn^–/–^* mice recapitulate several key features of PGRN-FTLD and NCL, including microgliosis, accumulation of lipofuscin, and retinal degeneration, which have been previously reported to be absent in *Grn^+/–^* mice (11, 12, 14, 15, 60). We therefore examined these phenotypes in 1-year-old *Nlk^+/–^ Grn^+/–^* mice to determine if loss of *Nlk* promotes pathology in the background of *Grn* haploinsufficiency.

**Figure 6:**
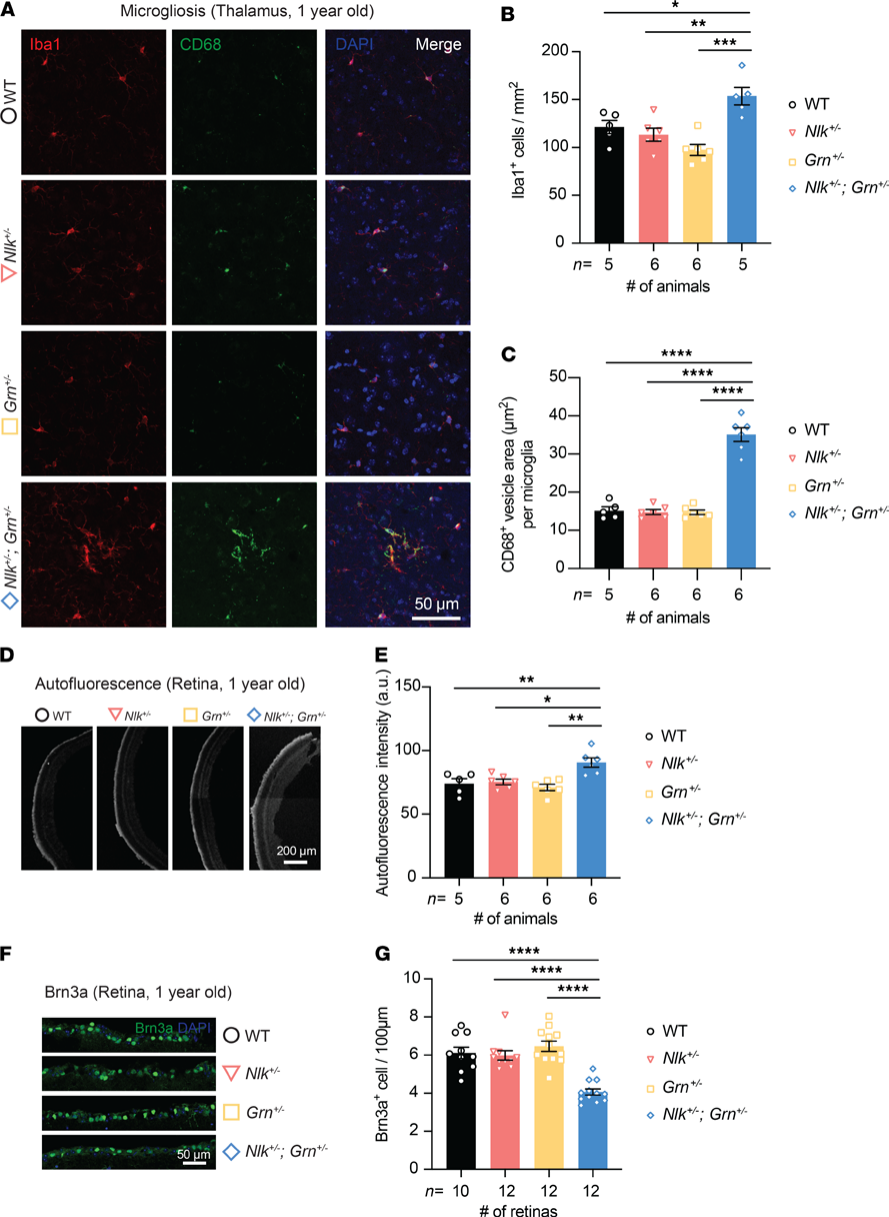
*Nlk^+/–^ Grn^+/–^* mice display FTLD-like neuropathological phenotypes. (**A–C**) Increased microglial activation in *Nlk^+/–^ Grn^+/–^* mice compared with their littermates. Representative confocal images (**A**) and quantification (**B** and **C**) of Iba1 and CD68 staining from the thalamus of 1-year-old mice. (**B**) The number of Iba1-positive microglia was increased in the thalamus of *Nlk^+/–^ Grn^+/–^* mice. *P < 0.05, **P < 0.01, ***P < 0.001; 1-way ANOVA with Tukey’s multiple comparisons post hoc test; *F*(3,18)=10.72, *P* = 0.0003. (**C**) CD68-positive vesicle volume in Iba1-positive microglia was also increased in *Nlk^+/–^ Grn^+/–^* mice. ****P < 0.0001; 1-way ANOVA with Tukey’s multiple comparisons post hoc test; *F*(3,19)=82.22, *P* < 0.0001. (**D** and **E**) Representative images (**D**) and quantification (**E**) of autofluorescence using 488 nm excitation in the retina of 1-year-old WT, *Nlk^+/–^*, *Grn^+/–^*, and *Nlk^+/–^ Grn^+/–^* mice. *P < 0.05, **P < 0.01; 1-way ANOVA with Tukey’s multiple comparisons post hoc test; *F*(3,19)=8.327, *P* = 0.0010. (**F** and **G**) Representative images (**F**) and quantification (**G**) of 1-year old WT, *Nlk^+/–^*, *Grn^+/–^*, and *Nlk^+/–^ Grn^+/–^* mouse retinas stained for Brn3a (green) and DAPI (blue). ****P < 0.0001; 1-way ANOVA with Tukey’s multiple comparisons post hoc test; *F*(3,40)=20.52, *P* < 0.0001.

To identify changes in microgliosis, ionized calcium-binding adapter molecule 1 (Iba1) and CD68 antibodies labeling microglia and microglial lysosomes, respectively, were used for immunohistochemistry. There was a significant increase in the number of Iba1-positive cells in the thalamus of 1-year-old *Nlk^+/–^ Grn^+/–^* mice compared with age-matched littermate controls (Figure 6, A and B). In addition, CD68-positive immunostaining showed increased lysosome volume in Iba1-positive microglia in 1-year-old *Nlk^+/–^ Grn^+/–^* mice (Figure 6, A and C). For both Iba1- and CD68-positive immunostaining, there was no significant difference between *Nlk^+/–^* and *Grn^+/–^* mice with WT littermate controls (Figure 6, A–C), further indicating that partial loss of *Nlk* and *Grn* contributes to microgliosis. These results are consistent with a previous report that showed microglia activation in *Grn*^–/–^ mice (26). To examine lipofuscin deposition, autofluorescence was assessed in the retina, thalamus, and cortex in 1-year-old mice. There was a significant accumulation of lipofuscin in the retina (Figure 6, D and E) and the thalamus (Supplemental Figure 4, A–C) of *Nlk^+/–^ Grn^+/–^* mice compared with littermate controls, and a trend toward lipofuscin accumulation in the cortex (Supplemental Figure 4, D and E). To examine retinal degeneration in this mouse model, immunohistochemistry was conducted for Brn3a, a transcription factor expressed in most retinal ganglion cells. We observed substantial degeneration of retinal ganglion cells in 1-year-old *Nlk^+/–^ Grn^+/–^* mice (Figure 6, F and G). Taken together, these neuropathological results show that Nlk may play an important role in modulating phenotypes typified by Pgrn reduction.

### Nlk reduction promotes behavioral alterations in Grn^+/–^ mice.

We next examined the effect of *Nlk^+/–^ Grn^+/–^* compound heterozygosity on mouse behavior. A recent report demonstrated that *Grn^–/–^* mice show disinhibition in the context of the elevated plus maze test (15), which is in agreement with FTLD patient behavioral changes (61). To determine if the genetic interaction between *Nlk* and *Grn* induces this behavioral phenotype in the mildly phenotypic *Grn^+/–^* mice, we assessed 1-year-old *Nlk^+/–^ Grn^+/–^* mice’s performance on the elevated plus maze (Supplemental Figure 5A). Since previous reports have shown genotype-dependent alterations in levels of anxiety specifically in male *Grn^–/–^* mice, only males were tested (62, 63). *Nlk^+/–^ Grn^+/–^* male mice were less anxious and disinhibited, as they spent more time in the open arms compared with littermate controls (Supplemental Figure 5A). *Grn^+/–^* and *Nlk^+/–^* mice alone did not have any significant behavioral phenotype relative to WT controls (Supplemental Figure 5A).

We also examined cognitive behavior by performing a Morris water maze (MWM) test at 1 year of age. *Nlk^+/–^ Grn^+/–^* mice had a significant impairment in identifying the hidden platform on trials 4–6 of testing relative to WT controls (Supplemental Figure 5B). As expected based on previous literature, *Grn^+/–^* mice performed similarly to WT controls (Supplemental Figure 5B). During the probe trial, *Nlk^+/–^ Grn^+/–^* mice showed a trend toward spending less time in the target quadrant than their WT littermates (Supplemental Figure 5C). To verify that these results were not an effect of alterations in locomotor behavior and visual acuity, swim speed and vision were also assessed (Supplemental Figure 5, D and E). There was no significant difference between *Nlk^+/–^ Grn^+/–^* mice and their littermates in swim speed across all 6 trials of MWM testing (Supplemental Figure 5D). Although we observed significant retinal degeneration in *Nlk^+/–^ Grn^+/–^* mice (Figure 6, F and G), this did not produce an observable behavioral deficit in vision, as there was no difference in the time spent to identify a visual cue (Supplemental Figure 5E). Collectively, these data indicate that *Nlk^+/–^ Grn^+/–^* mice have normal sensorimotor function. These results are correlated to the observed trending pathological phenotypes (Figure 6), as the thalamus, at least in part, contributes to the anxiety phenotypes and the cortex is important for the recognition phenotype. In addition, consistent with our pathological studies, these behavioral assays support the hypothesis that partial loss of *Nlk* and *Grn* additively or synergistically contributes to the appearance of pathological and behavioral phenotypes reminiscent to those reported in *Grn^–/–^* mice, although to a less severe extent.

## Discussion

Heterozygous and homozygous loss-of-function mutations in the *GRN* gene are common causes of familial FTLD and NCL, respectively (3, 5). Furthermore, reduced PGRN levels have been linked to other neurodegenerative disorders, including AD (8, 10, 64). Despite extensive studies, the biological and molecular mechanisms underlying Pgrn function and metabolism have yet to be completely understood. Furthermore, there is no cure or effective therapeutic to reverse or slow progression of PGRN-associated neurodegenerative diseases. This underlines the need for basic research to better understand Pgrn neurobiology and to identify effective strategies or proteins that could increase Pgrn levels in the brain, which will eventually be useful for the development of effective therapeutics for several neurodegenerative disorders associated with PGRN levels. In this study, we identified a novel mechanism in microglia through which overall brain PGRN levels can be controlled. We have found that altering the activity of the endocytosis/lysosomal pathway by modulating Nlk in microglia is sufficient to affect Pgrn levels and induce some pathophysiological phenotypes of *Grn* haploinsufficiency. By combining mouse genetic approaches and cell biological studies, we show that Nlk is a key protein regulating Pgrn levels in the brain via control of the receptor-mediated endocytosis/lysosomal pathway in microglia, and thus modulates the pathophysiology of *Grn* haploinsufficiency in the brain.

Contrary to *GRN* haploinsufficiency in humans, heterozygous loss of *Grn* in mice fails to produce robust neuropathological phenotypes central to FTLD-PGRN (11, 62). As a consequence, more attention has been directed toward characterizing mice that are null for *Grn*, which reproduces several features of human FTLD-PGRN and NCL (12, 14–16) and has enhanced our understanding of Pgrn function and biology in the brain, providing some fundamental insights into the pathophysiology of FTLD-PGRN. However, there are clear limitations to using *Grn*-deficient mice for such studies, one of which is whether and how increased expression levels of Pgrn would be able to rescue the phenotypes specific to FTLD-PGRN or NCL. Furthermore, the lack of overt neuropathological phenotypes in *Grn^+/–^* heterozygous mice or the absence of *Grn* gene and Pgrn protein in *Grn^–/–^* null mice lead to an unequivocal desire for the development and identification of more physiologically relevant animal models for FTLD-PGRN study to better understand the molecular and cellular mechanisms that contribute to neurodegeneration caused by *Grn* haploinsufficiency.

In this regard, it is very intriguing that this study shows that *Grn^+/–^* heterozygous mice develop several behavioral and neurodegenerative phenotypes, including cognitive impairments and altered anxiety, as well as lipofuscin accumulation, microgliosis, and retinal neurodegeneration, in the *Nlk^+/–^* heterozygous background, all of which are similarly observed in human patients with GRN haploinsufficiency (56, 65, 66). Consistent with previous studies, the *Grn^+/–^* heterozygous mice in the WT (*Nlk^+/+^*) background did not develop any phenotypes in the assays performed here (11, 62). To our knowledge, this is the first report showing that genetically heterozygous *Grn^+/–^* mice can develop relevant neurodegenerative pathological phenotypes in a certain genetic background, specifically a 50% Nlk expression reduction, which together leads to 70%–80% Pgrn reduction. As precise Pgrn dosage has been demonstrated to be crucial for proper nervous system function (7, 57), we expect that the presence of some remaining Pgrn in the compound heterozygotes would confer some advantage compared with the *Grn*-null mice, albeit an advantage insufficient to completely prevent the emergence of neurodegeneration-related phenotypes that we report here. While we have not conducted a side-by-side phenotypic comparison between *Nlk^+/–^* *Grn^+/–^* mice and *Grn^–/–^* null animals, we speculate that the reduction of Pgrn by Nlk haploinsufficiency in the *Grn* heterozygote lowers Pgrn protein levels to near threshold levels to induce some neurodegenerative phenotypes reminiscent of Pgrn deficiency and that perhaps a slight decrease in Pgrn dosage even further would generate significant pathological changes in all assessed brain regions.

The importance of expression levels and the functions of Pgrn in the brain, particularly in neurons and in microglia, have been well established (59, 67). A fundamental aim of these studies is to understand how Pgrn levels are regulated. In the brain, Pgrn protein is detected highly in neurons and activated microglia by immunostaining (35), while *Grn* mRNA is expressed highly in microglia and lowly in neurons and astrocytes (34). Conditional deletion of *Grn* specifically either in neurons or in microglia is not sufficient to cause phenotypes associated with Pgrn deficiency in mice (19, 20), which may be due to extracellular Pgrn levels being maintained at functionally sufficient levels by different cell types. It is curious that a 50% reduction of Nlk, which in combination with heterozygous deletion of *Grn* results in a net reduction of 70%–80% Pgrn levels through microglial regulation, is sufficient to induce neuropathological changes, while complete deletion of *Grn* gene in microglia is insufficient (20). A possible explanation to reconcile our potentially novel findings with previous reports is centered on active (e.g., producer) versus passive (e.g., modulator) reduction of Pgrn levels. In the case of conditional KO of *Grn* in microglia, microglia may be subject to constitutive compensation by other cell types, such as neurons, to maintain required Pgrn protein levels, which can be subsequently endocytosed by microglia to mitigate deleterious effects of the conditional KO. In the case of *Nlk* and *Grn* compound heterozygosity, the reduction of Pgrn levels is further exacerbated by active, enhanced, and constant catabolism of Pgrn protein through the endocytosis/lysosomal pathway in microglia, which is not the case in the models with genetic perturbations to *Grn* directly.

Our studies reveal 4 significant findings on the regulation of Pgrn levels in the brain: (a) Microglia regulate brain Pgrn levels; (b) Nlk is critical for the regulation of Pgrn levels in the brain; (c) Nlk regulates Pgrn levels via microglia, but not via neurons; and (d) Nlk-mediated control of Pgrn levels occurs at the level of protein, but not at the level of *Grn* mRNA expression. These findings support the main conclusion that Nlk controls Pgrn protein levels in the brain via microglia, which cannot be compensated for by other cell types. Consistent with this notion, Pgrn reduction levels (about 50% reduction) in the whole cortex of the microglia-specific KO (*Nlk^fl/fl^ Cx3cr1-cre*) mice are equivalent to those in Nlk deletion in all cell types (*Nlk^fl/fl^ Actin-cre^ERT2^*), suggesting that microglia are the major cells by which Nlk plays a fundamental role in regulating Pgrn levels in the brain.

Another important outcome of this study was revealing the cell biological mechanism underlying the Nlk-mediated regulation of Pgrn levels in microglia. These studies reveal that Nlk regulated Pgrn levels via control of the endocytosis/lysosomal pathways in microglia, but not in neurons. More specifically, Nlk negatively regulated microglial receptor-mediated (or clathrin-dependent) endocytosis, altering the delivery of multiple cargoes, including Pgrn, to the lysosome for subsequent proteolysis. Together, this information regarding the Nlk-mediated effect on the regulation of Pgrn levels in microglia reveals potentially new cell biological regulatory mechanisms of Pgrn catabolism and PGRN-associated disease pathogenesis, highlighting the significance of the present study.

The exact fate of lysosome-targeted Pgrn and the functional consequences of this increased processing in *Nlk^+/–^ Grn^+/–^* mice are not entirely clear, beyond the overall observation of the emergence of FTLD-related phenotypes. It is well appreciated that Pgrn undergoes a lysosome-dependent processing by cathepsins into stable granulin peptides (47, 68, 69), whose unique physiological roles in lysosome functioning have yet to be completely understood. Interestingly, Pgrn itself has been shown to interact with and affect the activity of lysosomal cathepsins (70, 71), suggesting an intricate homeostatic feedback between Pgrn and its processed forms with lysosomal enzymes. Future studies aimed at examining the effect of Nlk reduction on lysosomal function, especially in the context of Pgrn processing, could help to elucidate the pathological phenotypes we observe in *Nlk^+/–^ Grn^+/–^* animals.

Several important questions are raised by our study. First, what is the molecular basis of the Nlk-mediated cell type–specific regulation of endocytosis? In other words, why does Nlk regulate endocytosis specifically in microglia, but not in neurons, and what direct substrate(s) of Nlk control microglial endocytosis? Our preliminary studies suggest Nlk can phosphorylate several proteins involved in endocytosis, some of which are expressed specifically or very highly in microglia, but not in neurons. Second, which receptor(s) mediates Pgrn endocytosis in microglia? Previous studies have reported Sortilin and M6PR-LRP1-Prosaposin as 2 complementary pathways for delivery of Pgrn to the lysosome from extracellular space in other cell types (35, 36), suggesting that they may be modulated by Nlk to affect Pgrn trafficking. Third, because Nlk reduction affects clathrin-mediated endocytosis of multiple extracellular substrates in addition to Pgrn, what other proteins are impacted by extended modulation of Nlk levels? Fourth, what effect does Nlk have on Pgrn secretion? Finally, because Pgrn is processed into stable granulin peptides in the lysosome, which have been recently reported to have several detrimental (72) or beneficial (68) putative cellular functions, what effect does Nlk reduction have on the levels of individual granulins, and how might this affect overall lysosomal function? Although we were not able to reliably detect granulins in protein extracts used in this study (data not shown), follow-up studies using more sensitive immunoassays involving antibodies against specific granulin peptides (73–75) are warranted. Collectively, the findings of our study open the door for a more detailed mechanistic examination of the precise Nlk-mediated microglia-modulating effects, including characterization of the effects of varying Nlk levels on synaptic pruning and neuronal numbers in a brain circuit–specific manner, which may be involved in the behavioral changes we report here.

In conclusion, our study provides strong genetic and cell biological evidence that Nlk can control the microglial endocytosis/lysosomal pathway and modulate Pgrn levels and biology in the brain of a *Grn^+/–^* mouse model. It reveals a potentially new regulatory mechanism for the control of Pgrn levels and, consequently, its associated neurobiology in the brain, providing insight into the pathophysiology and the therapeutic development of PGRN-associated neurodegenerative diseases. This study also supports the future investigation of potential roles of Nlk and Nlk-mediated microglia modifications in diverse neurodegenerative diseases, including AD.

## Methods

### Mouse husbandry and genetics

Mice were maintained on a 12-hour light/12-hour dark cycle with standard mouse chow and water ad libitum. Two distinct *Nlk* gene trap (RRJ297 and XN619) mouse lines were maintained on a pure C57BL/6J background (48). To generate *Nlk* heterozygous pups for primary microglia culture, *Nlk* heterozygous (*Nlk^RRJ297/+^* or *Nlk^XN619/+^*; simply *Nlk^+/–^*) mice and wild-type (WT) mice were crossed. To generate *Nlk* mutant pups for primary neuronal culture, *Nlk* heterozygous (RRJ297/+ or XN619/+) mice were intercrossed. All 4 F1 progenies, including WT (*Nlk^+/+^*), heterozygous (*Nlk^RRJ297/+^* or *Nlk^XN619/+^*; collectively *Nlk^+/–^*), and compound heterozygous (*Nlk^RRJ297/XN619^*; simply KO here) pups, were used at postnatal day (P) 0. The *Nlk* conditional KO allele (*Nlk^fl/fl^*) mice were codeveloped with Ernesto Canalis (49) and deposited to the Jackson Laboratory (JAX #024537). *Nlk^fl/fl^* mice were backcrossed onto a pure C57BL/6J background over 10 generations before mating to *Actin-cre^ERT2^* (50), *Nex-cre* (51), or *Cx3cr1-cre* (53) mice. To activate the Cre in Cre^ERT2^, *Nlk^fl/fl^* *Actin-cre^ERT2^* and their littermate control mice received daily intraperitoneal injections of TMX (MilliporeSigma, T5648, 100 mg/kg) for 7 consecutive days at 6 weeks of age. *Grn^–/–^* mice were obtained from the Jackson Laboratory (JAX #013175). To perform the genetic interaction study, *Nlk^+/–^* heterozygote mice were bred to *Grn^+/–^* heterozygote mice, and all 4 subsequent F1 progeny (WT, *Nlk^+/–^*, *Grn^+/–^*, and *Nlk^+/–^ Grn^+/–^*) were obtained. Both male and female mice were used in this study unless mentioned otherwise.

### Mouse behavioral tests

#### Elevated plus maze.

Elevated plus maze was set at a height of 65 cm and consisted of 2 open white plexiglass arms, each arm 8 cm wide × 30 cm long and 2 enclosed arms (30 cm × 5 cm) with 15 cm high walls, which were connected by a central platform (5 cm × 5 cm). Individual mice were placed at the center of the maze, facing one of the closed arms, and observed for 5 minutes. All apparatus arms were cleaned with 70% ethanol after every trial. Data acquisition was recorded on a JVC Everio, G-series, camcorder, and analysis of time spent on open and closed arms was performed using Panlab’s Smart tracking and analysis program, v2.5.

#### Morris water maze test.

Twelve-month-old mice were tested in a cylindrical tank of 100 cm in diameter and 60 cm in height. The tank was filled with water at 25°C, and the platform was submerged 1 cm below the water surface. The tank was divided into 4 quadrants with different navigational landmarks for each quadrant. The midpoint of the wall in each quadrant was used as the starting location from which animals were released into the water. In the hidden platform acquisition test, mice were allowed to swim freely to search for the escape platform within 60 seconds. The platform location remained constant throughout the test. The time taken to reach the platform was recorded as the escape latency. The mouse was kept on the platform for 10 seconds after it found the hidden platform. If a mouse failed to find the platform within 60 seconds, it was guided to the platform and placed on the platform for 10 seconds; in this case, the escape latency was recorded as 60 seconds for this trial. The same animal was released from a new starting quadrant 4 minutes after the previous trial. The experiment was repeated with 6 trials per mouse, with 1 trial every morning and afternoon for 3 consecutive days. The mean escape latency was measured to evaluate the spatial learning ability.

Twenty-four hours after the hidden platform acquisition test, probe trials were conducted by removing the platform. Mice were placed in the quadrant opposite to the removed hidden platform and were allowed to swim freely in the pool for 60 seconds. The percentage of time spent in the area around the original hidden platform was used to indicate long-term memory maintenance. At the end of the probe trial, a colorful flag was placed on the top of the hidden platform, which was opposite to the testing quadrant. The mouse was released from 3 different quadrants 7 times, and the time spent to find the flag was recorded as a measure of visual ability. The probe trials were recorded on a JVC Everio, G-series, camcorder and analyzed using Panlab’s Smart tracking and analysis program, v2.5. The observer was blinded to genotype for the duration of behavioral testing.

### Western blot analysis

To examine protein expression levels, samples were prepared from the whole cortex of each mouse genotype or the cultured cells. Supernatant culture media from BV2 cells was collected for extracellular protein analysis as shown previously (76). To prepare intracellular protein samples, dissected mouse cortices were homogenized in PBS with a dounce homogenizer and centrifuged for 10 minutes at 3400*g* at 4°C. The pellet or harvested BV2 cells was lysed in lysis buffer (50 mM Tris [pH 7.5], 150 mM NaCl, 0.1% SDS, 0.5% Triton X-100, 0.5% NP-40, and Roche complete protease inhibitor cocktail), rotated at 4°C for 20 minutes, and then centrifuged for 10 minutes at 16,000*g* at 4°C. The supernatant was collected as the intracellular protein fraction. All samples were quantified and 20 or 40 μg total protein from each sample was boiled for 10 minutes in sample buffer (Bio-Rad 161-0737), loaded onto an SDS-PAGE gel, and transferred to a nitrocellulose membrane for Western blot analysis. Membranes were blocked by incubation in 5% skimmed milk powder in TBST, incubated with primary antibodies in TBST containing 5% skimmed milk, and then incubated in secondary antibodies conjugated with HRP, before detection with ECL reagents. The following primary antibodies were used: mouse anti-Vinculin (MilliporeSigma, V9264), sheep anti-Pgrn (R&D Systems, AF2557), rabbit anti-Nlk (Abcam, ab26050), mouse anti-Flag (MilliporeSigma, F3165), and rabbit anti-Flag (MilliporeSigma, F7425).

### Quantitative real-time reverse transcription polymerase chain reaction

RNA extraction, cDNA synthesis, and qRT-PCR were similarly performed as described (77). Total RNA was extracted from mouse cortices with the optional DNase digest step according to the manufacturer’s instructions (Qiagen, #74136). cDNA was synthesized using the iScript cDNA Synthesis Kit (Bio-Rad, #170-8891). qRT-PCR was performed using the C1000 Thermal Cycler and quantified using the CFX96 Real-Time System (Bio-Rad). TaqMan gene expression assays and the iQ supermix (Bio-Rad, #170-8862) were used for PCR amplification and real-time detection of PCR products. All RNA samples were analyzed in triplicate and normalized relative to *ACTB* expression levels. The following probes from Invitrogen were used: *Grn* (Mm00433848_m1), *Nlk* (Mm00476435_m1), and mouse *ACTB* (4352933E).

### Primary cortical neuron culture

Primary cortical neurons were prepared from neonatal pups at P0. Cerebral cortices were isolated and freed from meninges. Tissues were first digested with papain (Worthington, LS003126) and DNase I (MilliporeSigma, 10104159001) in HBSS (Gibco, 14170-112) for 30 minutes at 37°C, then washed 3 times with HBSS and triturated with fire-polished glass pipettes until single cells were obtained. Cell suspensions were then filtered through a 40 μm cell strainer and seeded at different densities according to the experimental design. Neurons were first plated in Neurobasal media (Gibco, 21103-049) supplemented with B27 (Gibco, 17504-044) and 1% FBS (Gibco, 16140-071) and changed to nonserum media 1 day later. Half media changes were performed every 3 days. Neurons were treated and collected at 7 to 8 days in vitro (DIV-7/8) for endocytosis analysis or collected at DIV-14 for protein and mRNA expression.

### Primary microglial cell culture

Primary cultured microglia were prepared from mouse brains, mainly from the cortex and the hippocampus, at P2 or P3. Meninges were removed mechanically, and the cells were dissociated by 0.25% trypsin (Gibco, 25200-056) in HBSS, then cultured in a poly-d-lysine–coated (PDL-coated) (MilliporeSigma, P6407) T25 flask with DMEM (Gibco, 11965-092) supplemented with 10% heat-inactivated FBS (65°C, 30 minutes) and penicillin/streptomycin. After 14 days (DIV-14), the culture flasks were shaken at 200 rpm for 3 hours to collect microglia. Microglia from 3 flasks having the same genotype were pooled and counted as 1 sample. In total, 3 independent experiments were repeated. All experiments were performed at DIV-16 to DIV-18.

### Generation of CRISPR-mediated Nlk-KD cells

The CRISPR/Cas9 technology (54) was used to generate *Nlk*-KD cells. Two guide RNAs were cloned separately into px462_v2 plasmid (Addgene, #62987, a gift from Feng Zhang, Massachusetts Institute of Technology, Cambridge, Massachusetts, USA). Then, 250 ng of each plasmid was cotransfected into the murine microglial cell line BV2 (a gift from Katerina Akassoglou, Gladstone Institutes, San Francisco, California, USA) using Nucleofector Kits (Lonza, VPI-1006). At 24 hours posttransfection, cells were enriched by the treatment of 4 μg/mL puromycin (Thermo Fisher Scientific, #A1113802) for an additional 48 hours. Living cells were dissociated and diluted to 1 cell/100 μL in DMEM and cultured in a 96-well plate to obtain a clonal population. Gene-targeting efficiency for each clone was analyzed by Western blot analysis. Due to the incomplete targeting of *Nlk* genes on the nondiploid BV2 cancer cell line, a single *Nlk*-KD BV2 cell clone was identified and selected for our analyses. The 2 guide RNA sequences used were #1, 5′-CCCATCCCCGGCACCGGGTC-3′, and #2, 5′-AACAACGGGTCCCAAATTGT-3′.

### Cell culture

The murine microglial BV2 cells were grown in DMEM supplemented with 10% heat-inactivated FBS and maintained at 37°C and 5% CO_2_. Cells were transiently transfected, using Amaxa Nucleofector (Lonza), according to the manufacturer’s instructions, with the *pcDNA3.1*, *Flag-mNlk-*WT, or *Flag-mNlk-*T298A constructs. For lysosomal-mediated degradation inhibition experiments, BV2 cells were grown in a 12-well plate to 70% to 80% confluence before treatment. Lysosomal degradation was blocked by BafA1 (LC Laboratories, B-1080, 400 nM) for 4 hours. To investigate the endocytosis-lysosome-mediated degradation of PGRN in WT or *Nlk-*KD BV2 cells, cells were treated first with BafA1 or DMSO for 4 hours, the culture media was removed, and cells were washed twice with PBS before treating with 20 nM of recombinant human PGRN (Flag-PGRN recombinant, AdipoGen, AG-40A-0068) for 15 minutes. For primary cortical neurons, the recombinant human PGRN incubation time was increased to 1 hour with a 20 nM concentration. For the clathrin-mediated endocytosis study, cells were treated first with Pitstop 2 (MilliporeSigma, SML1169, 30 μM) or DMSO for 1 hour; then the culture media were removed, and cells were treated with 1 nM of recombinant human PGRN for 15 minutes before collecting media.

### Dextran uptake

BV2 microglial cells were plated on 24-well plates with a round glass coverslip (Electron Microscopy Sciences, 72196-12) for 2 days prior to the experiment. The cells were placed on ice for 10 minutes, followed by incubation with Alexa Fluor 647–conjugated dextran (Molecular Probes, Thermo Fisher Scientific, D22914) at 1 mg/mL in DMEM for 20 minutes at 37°C. After washing twice with PBS, cells were fixed with 4% paraformaldehyde (PFA, MilliporeSigma, 158127) for 10 minutes.

### Transferrin uptake

BV2 cells, primary microglia, or primary cortical neurons were plated on PDL-coated glass bottom dishes (MatTek, P35G-1.5-14-C) 2 days before experiments. Cells were placed on ice for 10 minutes, followed by incubation with Alexa Fluor 568–conjugated transferrin (Molecular Probes, Thermo Fisher Scientific, T23365) at 25 μg/mL in Live Cell Imaging Solution (LCIS; Invitrogen, Thermo Fisher Scientific, A14291DJ) containing 20 mM glucose and 1% BSA for 15 minutes at 37°C. Cells were washed twice with cold LCIS before live cell image acquisition.

### Immunofluorescence staining

Immunofluorescence staining and confocal microscopy analyses were performed using frozen mouse tissues or cultured cells as described previously (48, 78). Briefly, mouse brains were carefully removed after cardiac perfusion, fixed overnight in 4% PFA, and incubated in 20% and 30% sucrose gradient per day at 4°C. Cultured cells were also fixed in 4% PFA for 10 minutes. Sectioned mouse brain slides or cells were permeabilized in PBS with 0.5% Triton X-100 and incubated with a blocking buffer (5% normal goat serum and 0.05% Triton X-100 in PBS), followed by primary antibody incubation in the blocking buffer at 4°C overnight and secondary antibody incubation for 2 hours. Brain sections were incubated with TO-PRO-3 iodide (642/661) (Invitrogen, Thermo Fisher Scientific, T3605) and mounted in Vectashield (Vector Laboratories, H1400). Cultured cells were directly mounted in Vectashield with DAPI (Vector Laboratories, H1500). The following primary antibodies were used in this study: sheep anti-Pgrn (R&D Systems, AF2557), rabbit anti-Iba1 (Wako, 019-19741), rabbit anti-Rab5 (Cell Signaling Technology, 3547), rabbit anti-Rab7 (Cell Signaling Technology, 2094), rabbit anti-Rab11 (Cell Signaling Technology, 5589), rat anti-CD68 (Abcam, ab53444), rat anti-Lamp1 (Developmental Studies Hybridoma Bank, 1D4B), and rat anti-Lamp2 (Developmental Studies Hybridoma Bank, ABL-93). Pgrn expression was detected in mouse brain sections with antigen retrieval (10 mM citrate pH 6.0 for 20 minutes at 90°C) before primary antibody was applied.

### Autofluorescence analysis

For autofluorescence analysis on mouse brains, sectioned tissues were washed in PBS. Without primary or secondary antibody incubation, sectioned brain tissues were coverslipped with Vectashield with DAPI and visualized on a Zeiss LSM710 Spectral confocal microscope with multiple excitation wavelengths including 488 nm and 543 nm. Images are *Z*-stack composites encompassing the entire section.

### Retinal ganglion cell counts

Retinas from both eyes were dissected and processed as described (15). Briefly, retinas were postfixed for 1 to 3 hours in 4% PFA, cryoprotected in 30% sucrose solution overnight at 4°C, embedded using OCT, and rapidly frozen on dry ice. Sections of 15 μm were sliced on a cryostat and collected directly onto microscope slides and stained. Primary antibody (mouse anti-Brn3a, Santa Cruz Biotechnology, 14A6, sc-8429) was applied overnight at 4°C. The secondary antibody used was Alexa Fluor 488–conjugated goat anti–mouse IgG (Invitrogen, A11001). TO-PRO-3 iodide (642/661) was used to stain nuclei. Fluorescent images were scanned using a Zeiss LSM710 Spectral confocal microscope with a 40× objective and processed with Zen 2.1 software (Carl Zeiss). Within the ganglion cell layer, the number of immunostained Brn3a cells was counted per 100 mm for each retinal section from the central regions. Data were averaged from 3 slices of each retina.

### Image acquisition and data analyses

For cultured cells, images were captured using a Zeiss spinning disk confocal microscopy (SDC) with a 60X oil objective lens or Zeiss LSM800 confocal microscope with a 63X oil objective lens. For mouse brain sections, images were captured using a Zeiss LSM710 Spectral confocal microscope with a 20X or 40X objective and processed with Zen 2.1 software (Carl Zeiss), followed by *Z*-stack composites encompassing the entire section. Three sections per animal were analyzed. Quantification of fluorescence intensity or endosomal/lysosomal puncta and colocalization was performed by Volocity software (PerkinElmer) or CellProfiler software (Broad Institute).

### Statistics

Data are presented as mean ± SEM. The statistical significance was assessed using *t* test, 1-way ANOVA, or 2-way ANOVA on the GraphPad Prism 6.0 software (GraphPad Software), as detailed in the figure legends for each panel. A value of *P* < 0.05 was considered statistically significant.

### Study approval

All animal experiments were approved by the Institutional Animal Care and Use Committee of Yale University and performed according to the National Institutes of Health guidelines for the care and use of laboratory animals.

## Author contributions

TD, LT, and JL conceived the project. TD, LT, YJ, HK, and KL performed experiments. TD, LT, YJ, HK, KL, TMD, and JL analyzed and interpreted data. TD, LT, YJ, and JL wrote the manuscript. All authors reviewed the manuscript and discussed the work.

## Supplementary Material

Supplemental data

## Version 1 10/07/2021

In-Press Preview

## Version 2 11/22/2021

Electronic publication
