# Supplementary material for: Microglia regulate brain progranulin levels through the endocytosis/lysosomal pathway
Source: JCI Insight. 2021 Nov 22;6(22):e136147. doi: 10.1172/jci.insight.136147 (PMC8663778; doi:10.1172/jci.insight.136147)
Supplement: Supplemental data [file jciinsight-6-136147-s151.pdf]

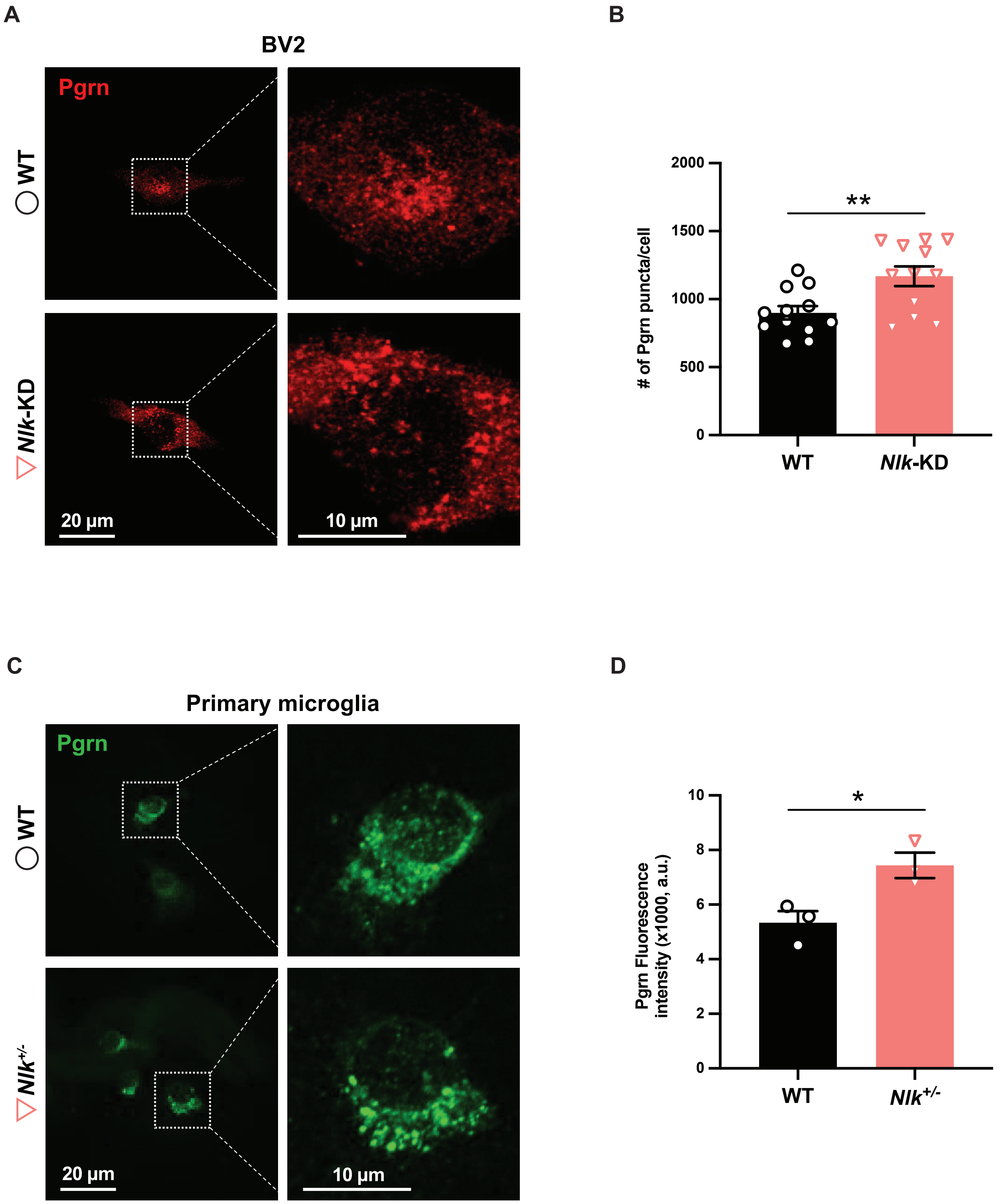

Dong et al._Figure S1

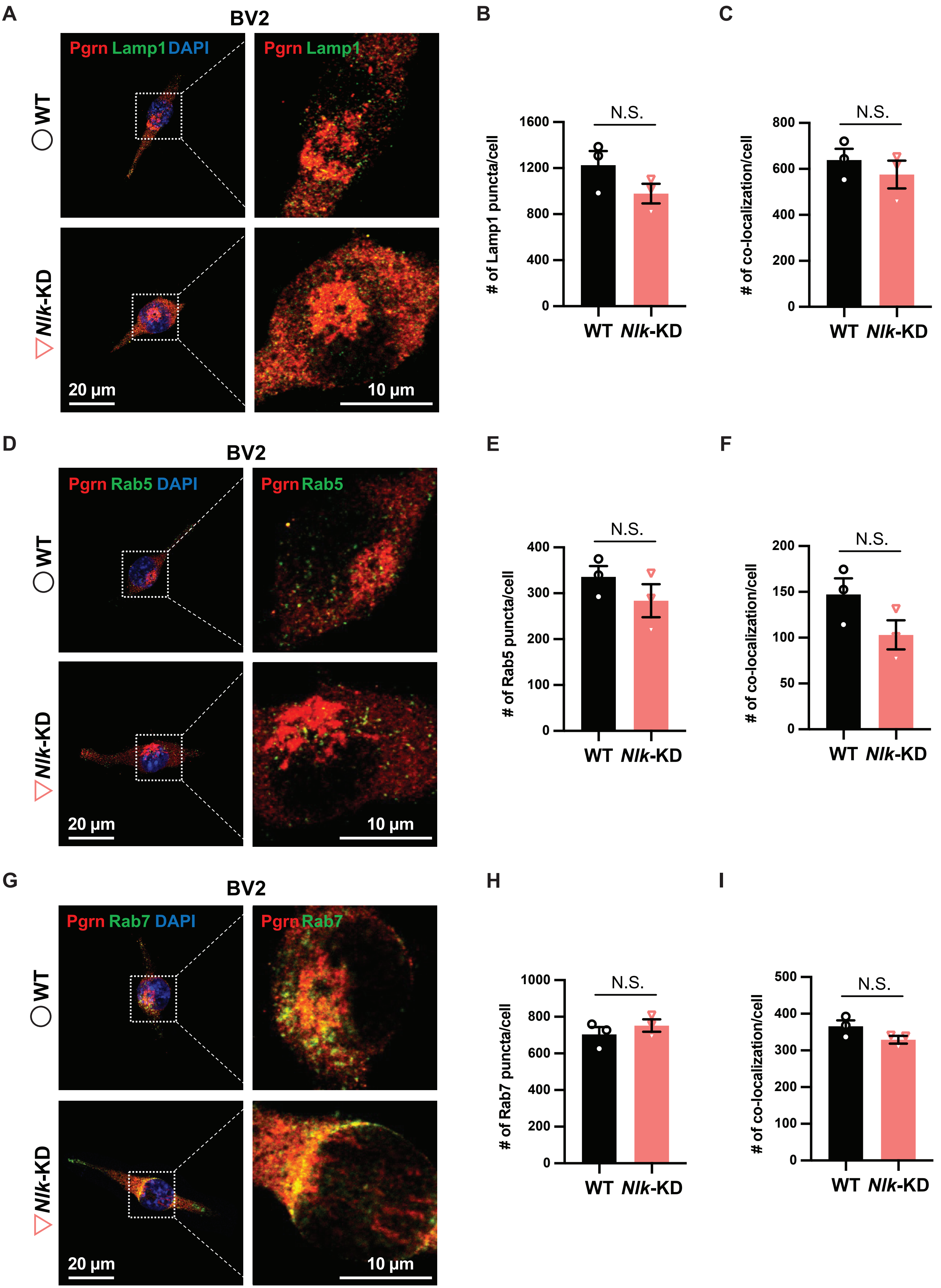

Dong et al._Figure S2

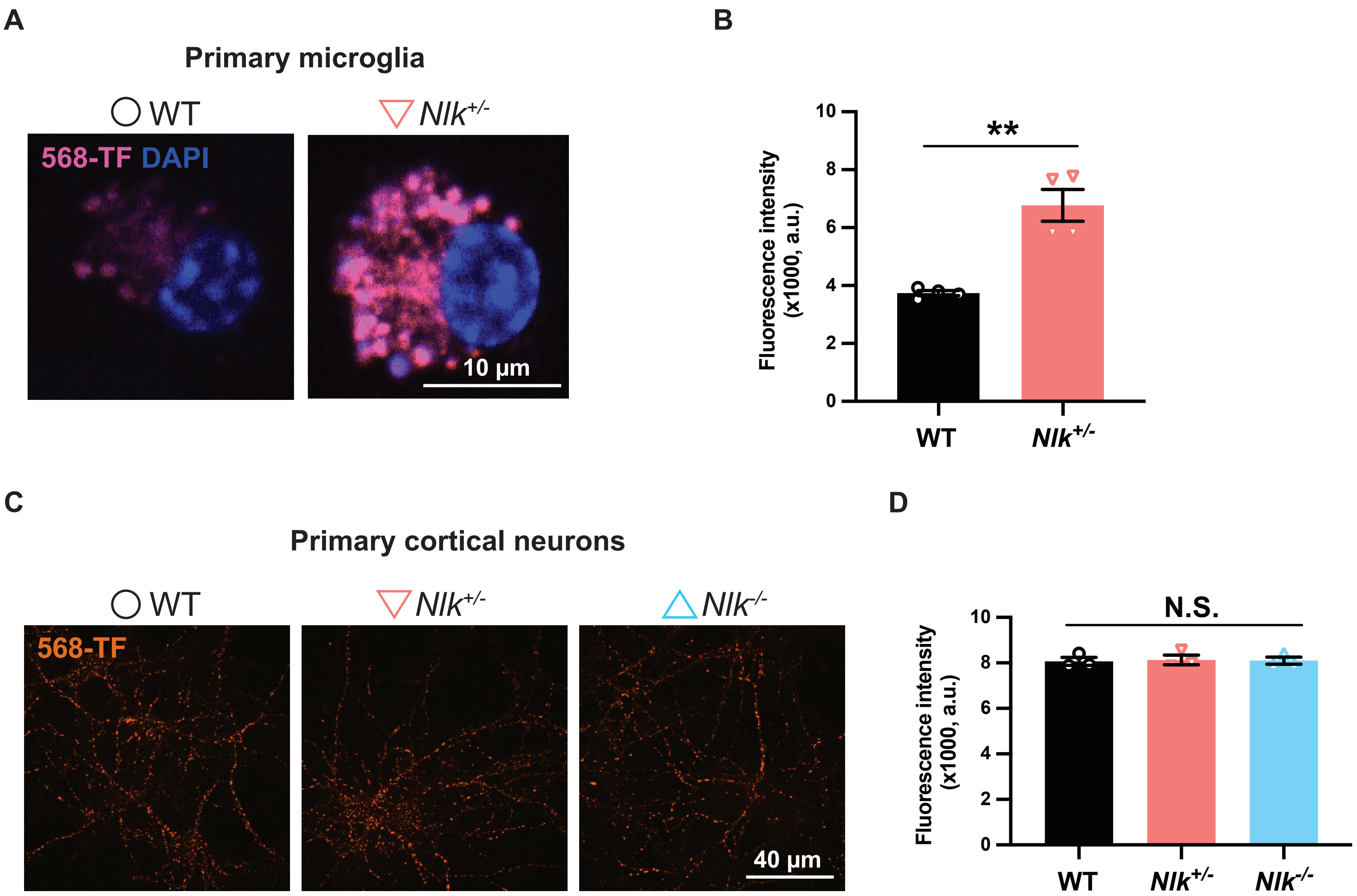

Dong et al._Figure S3

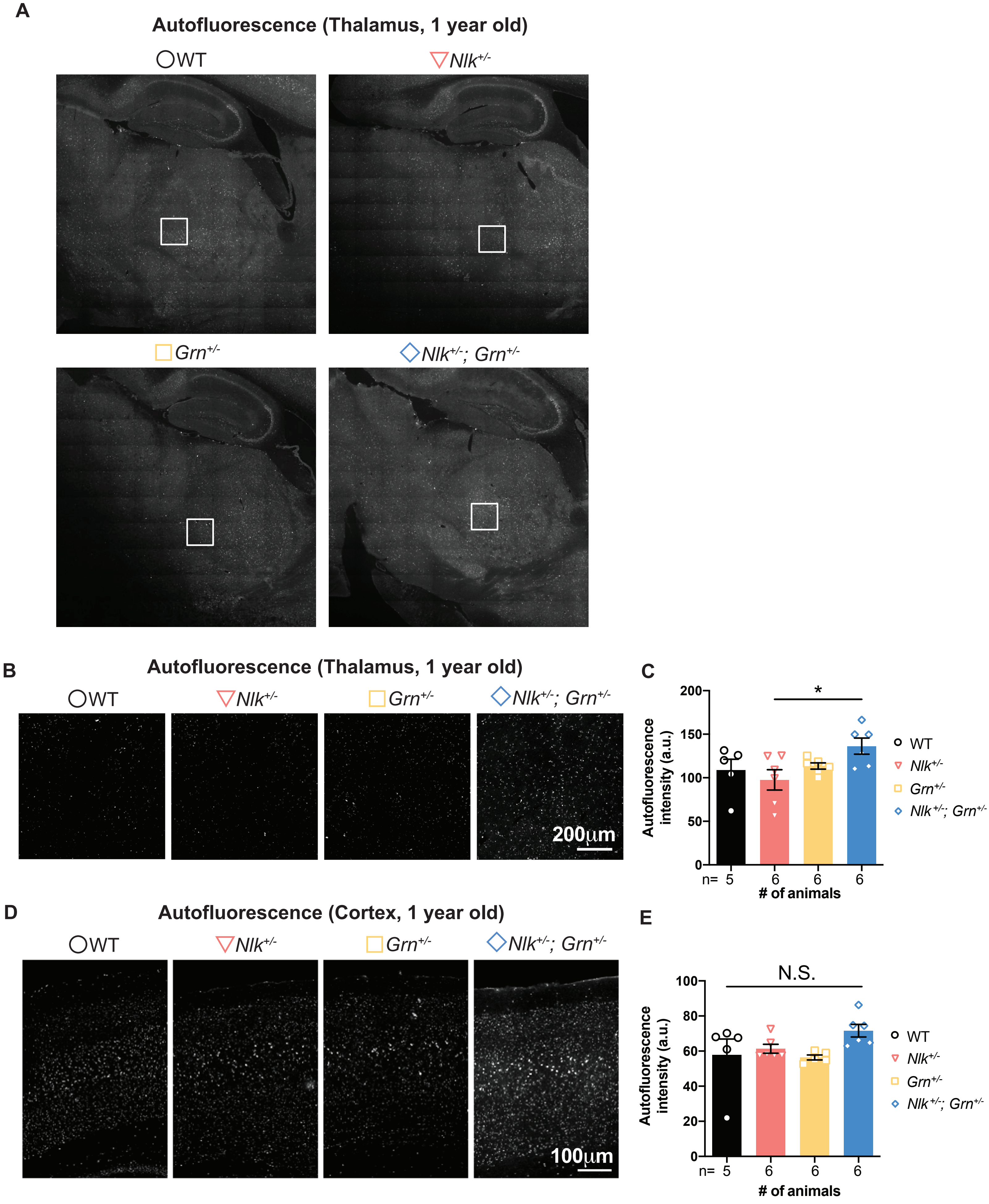

Dong et al._Figure S4

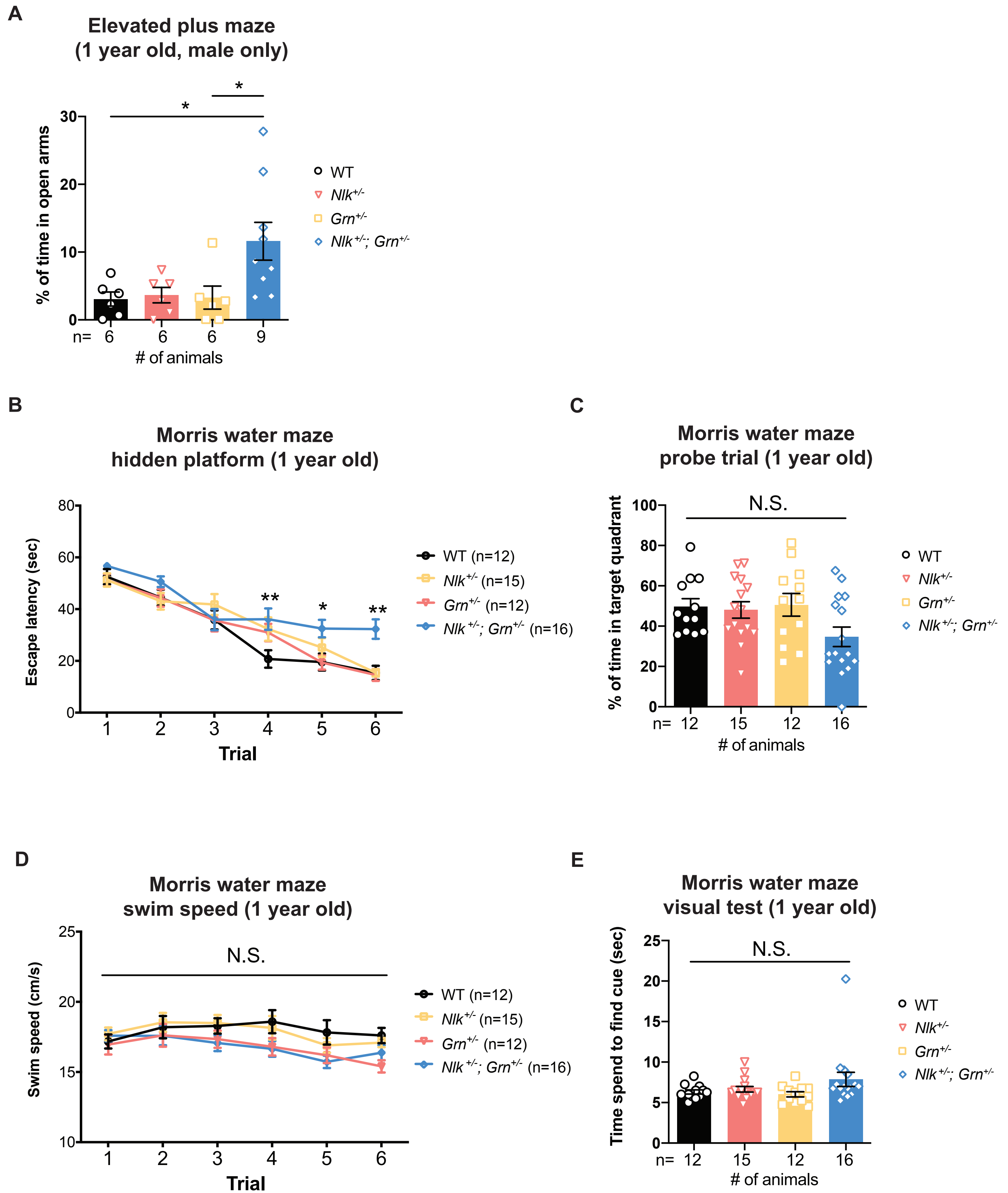

Dong et al._Figure S5
